# Exercise training reduces sympathetic nerve activity and improves executive performance in individuals with obstructive sleep apnea

**DOI:** 10.6061/clinics/2021/e2786

**Published:** 2021-08-23

**Authors:** Thiago Tanaka Goya, Rosyvaldo Ferreira-Silva, Elisangela Macedo Gara, Renan Segalla Guerra, Eline Rozária Ferreira Barbosa, Edgar Toschi-Dias, Paulo Jannuzzi Cunha, Carlos Eduardo Negrão, Geraldo Lorenzi-Filho, Linda Massako Ueno-Pardi

**Affiliations:** I Intituto do Coracao (InCor), Hospital das Clinicas HCFMUSP, Faculdade de Medicina, Universidade de Sao Paulo, Sao Paulo, SP, BR.; II Escola de Artes Ciencias e Humanidades, Universidade de Sao Paulo, Sao Paulo, SP, BR.; III Nucleo de Apoio a Pesquisa em Neurociencia Aplicada (NAPNA), Universidade de Sao Paulo, Sao Paulo, SP, BR.; IV Departamento de Psiquiatria, Hospital das Clinicas HCFMUSP, Faculdade de Medicina, Universidade de Sao Paulo, Sao Paulo, SP, BR.; V Escola de Educacao Fisica e Esportes, Universidade de Sao Paulo, Sao Paulo, SP, BR.

**Keywords:** Exercise Training, Sympathetic Nervous System, Executive Function, Obstructive Sleep Apnea

## Abstract

**OBJECTIVE:**

To investigate the effects of exercise training (ET) on muscle sympathetic nerve activity (MSNA) and executive performance during Stroop Color Word Test (SCWT) also referred to as mental stress test.

**METHODS:**

Forty-four individuals with obstructive sleep apnea (OSA) and no significant co-morbidities were randomized into 2 groups; 15 individuals completed the control period, and 18 individuals completed the ET. Mini-mental state of examination and intelligence quotient were also assessed. MSNA assessed by microneurography, heart rate by electrocardiography, blood pressure (automated oscillometric device) were measured at baseline and during 3 min of the SCWT. Peak oxygen uptake (VO_2_ peak) was evaluated using cardiopulmonary exercise testing. Executive performance was assessed by the total correct responses during 3 min of the SCWT. ET consisted of 3 weekly sessions of aerobic exercise, resistance exercises, and flexibility (72 sessions, achieved in 40±3.9 weeks).

**RESULTS:**

Baseline parameters were similar between groups. Heart rate, blood pressure, and MSNA responses during SCWT were similar between groups (*p*>0.05). The comparisons between groups showed that the changes in VO_2_ (4.7±0.8 *vs* -1.2±0.4) and apnea-hypopnea index (-7.4±3.1 *vs* 5.5±3.3) in the exercise-trained group were significantly greater than those observed in the control group respectively (*p*<0.05) after intervention. ET reduced MSNA responses (*p*<0.05) and significantly increased the number of correct answers (12.4%) during SCWT. The number of correct answers was unchanged in the control group (*p*>0.05).

**CONCLUSIONS:**

ET improves sympathetic response and executive performance during SCWT, suggesting a prominent positive impact of ET on prefrontal functioning in individuals with OSA. ClinicalTrials.gov: NCT002289625.

## INTRODUCTION

Obstructive sleep apnea (OSA) is characterized by repetitive episodes of partial (hypopnea) or total (apnea) obstruction of the upper airways during the sleep (1). Growing evidence shows that OSA is associated with cardiovascular disease (2) and cognitive deficits (3). In addition, individuals suffering from OSA have increased morbidity and mortality (4,5).

Sympathetic hyperactivation at rest and during sleep has been reported in individuals with OSA (6). Hypoxia and hypercapnia, markers of OSA, activate chemoreceptors, which in turn, increase reflexes in muscle sympathetic nerve activity (MSNA). Reduction of baroreflex mediating suppression of MSNA (7) and changes in central mechanisms (8) also contribute to the increase in MSNA. In individuals with OSA, increased MSNA and arterial blood pressure (BP) were associated with a higher intensity of the functional magnetic resonance signal in the prefrontal cortex region (8). This area is involved in sympathetic outflow and executive functioning, including inhibitory control and attention as well (9). Cognitive declines are present in subjects with OSA, mainly impacting executive function, which involves the ability to perform tasks, such as planning, cognitive strategies, organization, inhibitory control, decision making, problem solving, and attention-seeking processing effort (10-12). According to our previous work (9), significant correlations were observed between sleep parameters and executive function as measured by the Stroop Color Word Test (SCWT) in individuals with moderate to severe OSA.

Sympathetic nerves influence the ability of cerebral capacitance vessels to regulate intracranial pressure and cerebral blood volume (13). Sympathetic nerves play a role in physiological homeostasis of cerebral vessels, and OSA that alters sympathetic activity would be likely to alter this control. The higher sympathetic activity linked to abnormal cerebral vascular regulation could perhaps be a possible precursor to cognitive dysfunction and other pathology in the cerebral vascular system (14). In fact, a decrease in cerebral blood flow at rest was associated with worsening of cognition in individuals with OSA (15), which may indicate a potential association between sympathetic hyperactivity and loss of executive performance in these individuals.

Exercise training (ET) has been thought to play an important role in the prevention and treatment of chronic diseases, by promoting a reduction in sympathetic outflow in hypertensive and heart failure patients (16,17). In addition, ET has been shown to improve cognitive functioning (18) and sleep parameters in individuals with sleep apnea (17,19), but the interaction between neural measures and executive functioning among individuals with moderate to severe OSA remains poorly understood.

The aim of the present study was to investigate the effects of ET on MSNA and executive performance during SCWT. We hypothesized that ET would reduce MSNA levels during mental challenges and improve executive performance during SCWT in individuals with moderate to severe OSA. In addition, we sought to verify whether a correlation exists between MSNA and executive function after ET.

## METHODS

### Population

Male and female individuals, 40 to 65 y of age, were recruited from the community enriched by relatives and friends from the staff of the Heart Institute Hospital. These participants were part of a large study regarding ET on cerebral function in individuals with OSA. Of note, some study subjects took part in our previous dealing with the effects of ET on muscle metaboreflex control and cardiac autonomic modulation (20,21).

Exclusion criteria were treated OSA, sleep disturbance other than OSA, shift workers, body mass index >40 kg/m^2^, hypertension, cardiovascular and cardiopulmonary diseases, chronic kidney disease, diabetes mellitus, left ventricular ejection fraction <45%, psychiatric and neurodegenerative disorders, dementia, smoking or alcohol abuse (2 or more drinks per day), less than 2 y of formal education, and use of chronic medication. The study was approved by the Research Committee of the Heart Institute (InCor) (SDC 3536/10/125) and Clinical Hospital, Faculty of Medicine, University of São Paulo (0833/10). This present study is registered at ClinicalTrials.gov (NCT002289625). Written informed consent was obtained from all participants.

### Experimental Design

All participants were sedentary adults who had not exercised regularly for at least 3 months before entering this study. Non-menopausal women were studied between the first and the fifth days after the start of menstruation. All subjects underwent evaluation of blood profile, echocardiography, Mini-Mental State Examination, Intelligence Quotient (IQ), using Wechsler Abbreviated Scale for Intelligence - WASI, Beck Depression Inventory, Beck Anxiety Inventory, and Epworth Sleepiness Scale questionnaires at baseline as described previously (9). Full nocturnal polysomnography, microneurography at rest and during SCWT, and maximal exercise capacity were performed at baseline and at the end of the study. After entering the study, the control group was monitored (clinical follow-up) and instructed to avoid exercise. Throughout the study, the subjects in the control group were frequently contacted to check for changes in the level of physical activity or medication. Individuals who started any continuous medication or treatment for sleep apnea were excluded.

### Sleep Study

Overnight conventional polysomnography (Embla N7000, Medcare Flaga, Reykjavik, Iceland) was performed on all individuals using the following parameters: electroencephalography, electrooculogram, submentonian, and anterior tibial electromyography, snoring sensor, air flow and nasal pressure cannula, thoracic and abdominal belts, electrocardiogram, position detector, finger oxygen saturation. Sleep stage was scored manually, and apneas and hypopneas were scored according to previously described methods (21). OSA was defined as a cessation of respiratory airflow for 10 sec with thoracoabdominal effort, which was detected by using a respiratory effort sensor.

### Cardiopulmonary Exercise Test

The maximum exercise capacity estimated by the VO_2_ peak was determined by ergospirometry, according to well established methods (21).

### Muscle Sympathetic Nerve Activity

MSNA was directly recorded from the peroneal nerve (leg) using microneurography (662C-4 Nerve Traffic Analysis System, The University of Iowa, Iowa City, IA, USA), according to well established methods (20,22). Muscle sympathetic bursts (bursts per min), and bursts per 100 heartbeats, were identified by visual inspection conducted by a single investigator, blind to the status of study participants (control *vs* exercise-trained).

### Hemodynamic Measures

Heart rate was assessed beat by beat using an electrocardiogram. BP was monitored with an automated device (DX 2022, Dixtal Biomedics, Manaus, AM, Brazil). Systolic and diastolic BP values were recorded every min during the SCWT.

### Stroop Color Word Test

A modified version of the SCWT was applied for a period of 3 min after a 4 min resting baseline. During the SCWT, the participants visualized a chart with the name of colors written in colored ink different from the meaning of the word. The subjects were asked to identify the color of the ink, and not to read the written words. Throughout the SCWT, the subjects were also instructed to answer as quickly as possible, if it took more than 3s to respond. At the end of the protocol, each participant was asked to evaluate the perception of stress using a scale from 0 to 5 (0 not stressful, 1 very mildly stressful, 2 mildly stressful, 3 moderately stressful, 4 very stressful, and 5 extremely stressful), as described previously (9).

### Exercise Training

The ET consisted of 72 sessions, 3 days per week. Exercise session included 5 min of stretching exercises, 40 min of cycling on an ergometer bicycle, 10 min of strengthening exercises, and 5 min of cool down. The cycling exercise intensity was established by heart rate levels that corresponded to an anaerobic threshold up to the respiratory compensation point obtained in the maximal cardiopulmonary exercise test. The strengthening exercise was performed at mild to moderate (5-7) exercise intensity, which consisted of dynamic exercises including the lumbar muscles, abdominals, quadriceps, hamstrings and triceps sural, upper back, lower back, pectorals, and deltoid muscles. The intensity was controlled by a perceived exertion scale ranging from 0 to 10, as reported previously (20).

### Statistical Analysis

The data are presented as mean±standard deviation. Baseline data were used to compare differences between control and exercise-trained groups using χ^2^, unpaired Student *t* tests or Mann-Whitney *U* tests. Repeated-measures ANOVA (2-way) was used to compare within and between group differences during the SCWT protocol. In the case of significance, post hoc comparisons were performed. Pearson’s correlation coefficient was used to investigate if changes in MSNA after ET correlated with changes in executive functioning during SCWT in trained individuals with OSA. A *p-*value of ≤0.05 was considered statistically significant. All analyses were performed using STATISTICA 12 software (StatSoft Inc., Tulsa, OK).

## RESULTS

### Baseline measures

**Figure 1:**
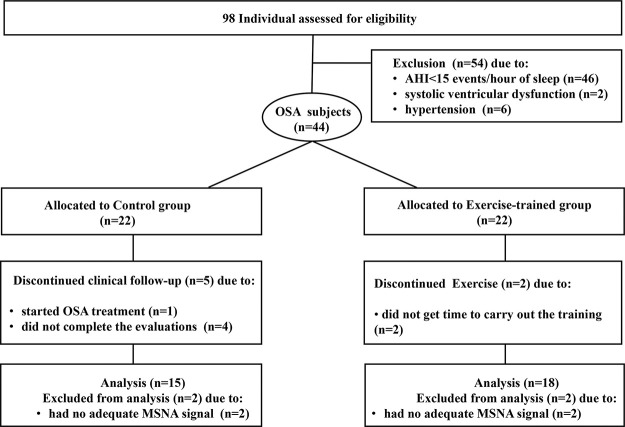
Profile of a randomized clinical trial showing the progress of individuals throughout the trial. AHI = apnea-hypopnea index; OSA = obstructive sleep apnea; MSNA = muscle sympathetic nerve activity.

From a total of 98 initially selected subjects potentially eligible to participate in the study, 54 subjects were excluded, due to no moderate to severe OSA (n=46); 2 subjects due to the presence of asymptomatic systolic ventricular dysfunction; 6 subjects had blood pressure (BP) ≥140/90 mm Hg. Figure 1 presents the profile of the randomized clinical trial showing the evolution of individuals throughout the trial. Forty-four subjects with a recent diagnosis of OSA (AHI> 15 events per hour of sleep) were included in the study. They were randomly assigned to the control group (n=22) and the exercise-trained group (n=22) through the recruitment sequence at the ratio of 1:1 in which one subject was selected for the control group and the next subject selected for the exercise-trained group. In the control group, 1 subject started sleep apnea treatment with the use of continuous positive airway pressure (CPAP), 2 subjects had no adequate signal of MSNA during SCWT, and 4 did not finish the entire protocol. In the exercise-trained group, 2 subjects did not follow the ET protocol, and 2 subjects had no adequate MSNA signal during SCWT. The baseline sociodemographic and clinical characteristics of the control and exercise-trained groups are shown in Table 1. No significant baseline differences existed between groups. Executive performance, indicated by the total number of correct answers during the 3 min SCWT, did not differ between control and exercise groups (115.6±9.0 *vs* 120±8.5, *p*>0.05).

### Effects of Exercise Training

Compliance with the exercise program (72 sessions or 100% of training) ranged from 85% to 100% of the exercise sessions attended for individuals with OSA. Seventy-two sessions of ET as planned was achieved in 40±3.0 weeks, whereas the control group remained without regular exercise for a total of 37±2.0 weeks. ET did not change body weight and BMI (Table 2). However, the group comparison showed that the change in body weight in the exercise-trained group was significantly greater than that observed in the control group (*p*<0.05).

**Figure 3:**
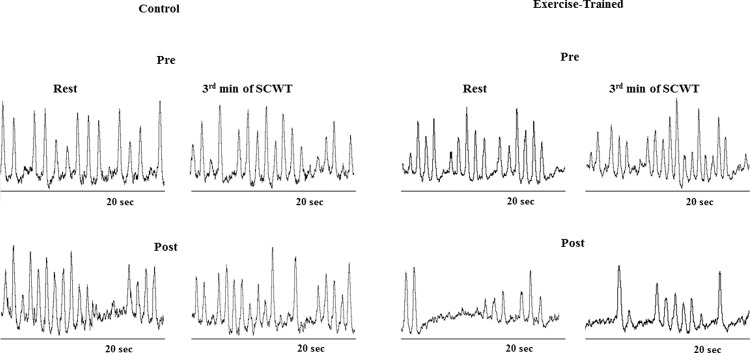
Sympathetic neurograms (30 sec) at rest and during 3 min of Stroop Color Word Test (SCWT).

MSNA increased during the second and third min of SCWT compared with the resting value before and after intervention/control (Figure 2A). ET significantly decreased MSNA levels at rest and during SCWT. No changes at rest or during SCWT were observed in MSNA in the control group after clinical follow-up. Analysis of delta responses (each minute of SCWT - baseline) also showed that MSNA increased in exercise-trained and control groups in the second and third min of SCWT, and these increases were similar before and after intervention/control (Figure 2B). Examples of sympathetic neurograms (30 sec) are shown in Figure 3. There were no differences (*p*>0.05) in perception of stress during SCWT between- or within-group comparison (pre control=2.9±0.4 *vs* pre exercise-trained 2.7±0.4), and (post control=2.9±0.3 *vs* post exercise-trained 2.6±0.4).

**Figure 4:**
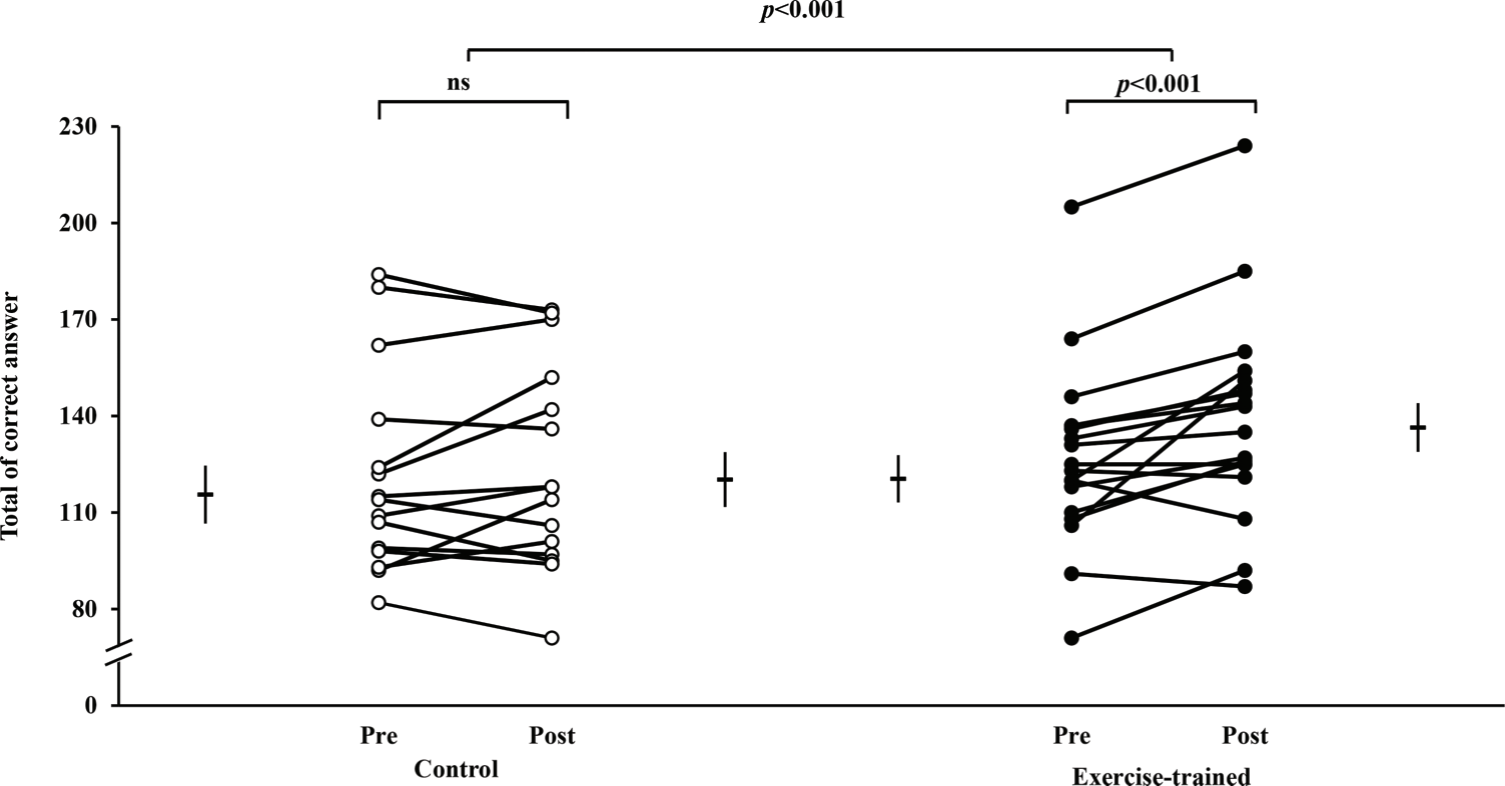
Individual values of executive performance indicated by the total number of correct answers during the SCWT. Short horizontal lines and bars are mean±SD. In the control group, the total number of correct answers from pre to post was similar (*p*>0.05). In contrast, the total number of correct answers significantly increased in the exercise-trained group. There were group x time interaction effects (*p*<0.001). SCWT = Stroop Color Word Test; ns = not significant.

Individual values of executive performance indicated by the number of correct answers during the SCWT are shown in Figure 4. ET significantly increased the number of correct answers during SCWT (120.5±7.3 *vs* 136±7.6). The number of correct answers was unchanged (115.6±9.0 *vs* 120±8.5) in the control group. The executive performance improved significantly only in the exercise-trained group and did not have a significant correlation with changes in MSNA (*p*>0.05).

HR significantly increased during 3 min of the SCWT compared with the baseline value before and after intervention/control (Figure 5A). Heart rate during the first min of SCWT was higher compared with heart rate in the second and third min of SCWT before and after intervention/control. Figure 5B shows delta response of the heart rate during the SCWT. The delta response of heart rate did not change after ET and clinical follow-up.

The mean BP significantly increased during the second and third min of SCWT compared with the baseline value before and after intervention/control (Figure 5C). The mean BP during the second min (both groups) and third min (exercise-trained group) of SCWT was also higher compared with mean BP in the first min of SCWT before and after intervention/control. Analysis of delta responses in mean BP during SCWT (Figure 5D) showed no significant changes before and after intervention/control.

ET significantly increased peak VO_2_ (*p*<0.05). No significant changes in peak VO_2_ were found in the control group (Table 2). Concerning sleep parameters, there were no changes in total sleep time and sleep efficiency between groups after intervention or clinical follow-up. However, ET significantly decreased the arousal index. The comparisons between groups also showed that the changes in arousal index, AHI, and O_2_ desaturation events in the exercise group were significantly greater than those observed in the control group (*p*<0.05).

## DISCUSSION

The main findings of the present study is first that ET reduces MSNA at rest and during SCWT; second it improves executive performance during SCWT demonstrated by an increase in the total number of correct answers during SCWT, and third no significant association was found between MSNA and executive functioning during SCWT in trained individuals with moderate to severe OSA.

OSA is associated with increased daytime and nocturnal sympathetic activity, which contributes to the increase in cardiovascular risk (23). The remarkable finding of the present study is the fact that ET decreased the levels of MSNA in individuals with moderate to severe OSA. Our results are in line with previous studies that showed that ET reduces resting MSNA in OSA (24) as well as other pathologies, including systolic heart failure (22) and myocardial infarction (25). In the present study, ET reduced MSNA (∼28.6%) towards levels lower than those observed in subjects with no OSA (∼28 bursts/min) (9). During SCWT, the tachycardia, elevation in BP, and increase in MSNA represent the typical fight or flight response. In the present study, the SCWT elicited moderate stress in control and exercise-trained groups. Our findings show that ET does not significantly influence the heart rate and BP response. Moreover, they provide evidence that ET reduces levels of MSNA during SCWT in individuals with OSA, which seems to be a consequence of a decrease in resting MSNA.

The mechanisms by which ET improves MSNA are multiple. A recent study demonstrated that ET improved both baroreflex and chemoreflex control in individuals with OSA and metabolic syndrome (24,26). In rats with heart failure, ET improved when the rats were given an angiotensin II, nitric oxide (27), and the neurotransmitter γ-aminobutyric acid (28) that act within the paraventricular nucleus participating in the neural control of the peripheral chemoreflex of MSNA.

ET significantly reduced the level of MSNA at rest and during SCWT without changes in mean BP. Potential mechanisms underlying the MSNA and BP responses to ET remain unclear. However, an increase in neurovascular transduction in exercise-trained OSA, as observed in the present study, may have happened, which helps to explain that the reduction in MSNA did not translate to a reduction in BP in response to SCWT. This increase in neurovascular transduction possibly could protect exercise-trained OSA against the deleterious effects of hypoxia on the cardiovascular system. Alternatively, ET may also reset the baroreflex control of the sympathetic vasomotor outflow. The effect of this resetting of the baroreflex control is that the arterial pressure is regulated around a level that is appropriate for the particular behavioral condition (29). The sympathoexcitation during mental tasks is associated with the development and progression of cardiovascular diseases (30). Thus, the improvement in MSNA levels during SCWT may be interpreted as cardiovascular protection particularly in individuals with OSA at increased risk for cardiovascular complications.

In the present study, we found that ET reduces MSNA levels and improves executive performance during SCWT. ET was able to improve (12.4%) executive performance during SCWT in individuals with OSA. The present study also addresses the possibility of ET reducing sympathetic activity and, consequently, improving executive function.

The tone of cerebral vessels and cerebral blood flow depend on cardiac output and the activity of sympathetic neurons that innervate cerebral blood vessels. Activation of sympathetic nerve fibers results in vasoconstriction that may extend to cerebral sympathetic nerves through modulation of cerebrovascular autoregulation and cerebral blood flow (31). The sympathetic nerve is responsible for alertness. During cognitive tasks, when there is an increased energy demand, regional cerebral blood flow is locally adjusted in the brain to meet this demand (32,33). Because there is sympathoexcitation in individuals with OSA (6), it seems plausible that an exaggerated sympathetically mediated cerebral vasoconstriction might underlie OSA decreases in cerebral blood flow possibly impacting neurovascular coupling and cognitive function. However, the extent to which sympathetically mediated cerebral vasoconstriction occurs during SCWT in individuals with OSA may be attenuated with our ET. A previous study reported that ET including aerobic exercise may help increase cerebral blood volume (34) and number of synapses (35) that are potentially beneficial to improve executive performance. These factors, therefore, may contribute to explain the benefits of ET over inhibitory functioning and attention during SCWT in individuals with OSA. In the present study, no significant association was found between changes in MSNA and changes in executive functioning during SCWT in trained individuals with moderate to severe OSA. Because this association was not observed, these potential mechanisms were not supported by the study results. It seems that more studies are needed with neuroimaging techniques to explore the cerebral vascular system, cerebral metabolic demand, using pharmacological blockade of adrenergic agents during SCWT in individuals with OSA to better understand the interaction between ET, sympathetic activity, and executive functioning in this group. This is an important topic for future research.

Our study has potential limitations. The present study was planned to carry out 6 months of supervised ET performed 3 times a week, totaling 72 training sessions. Unlike planned, the exercise-trained individuals were able to participate in the training with a frequency ranging from 1 to 3 times a week, which is something that happens in real life. Thus, we decided to extend the duration of the ET protocol to reach 72 sessions with the targeted heart rate training. However, in the present study, 72 exercise sessions, regardless of frequency, over a period of approximately 40 weeks, caused a significant improvement in functional capacity and executive function, reduced MSNA levels in exercise-trained individuals with OSA. Another limitation of this study is the reduced number of participants with OSA in the study. These factors can influence the degree of significance of some results. However, important strengths of our study are that all participants had nontreated OSA and were free of medications. The groups were matched for age, obesity, and physical activity status.

In conclusion, ET is effective in improving executive performance and reducing MSNA at rest and during SCWT in individuals with moderate to severe OSA. These responses are suggestive of better neural prefrontal control, as well as reduced cardiac risk during tasks with greater cognitive demand in this group.

## AUTHOR CONTRIBUTIONS

Goya TT contributed to scientific content, technical procedures, acquisition, and interpretation of the data. Ferreira-Silva R, Gara EM, Guerra RS, Barbosa ERF, Cunha PJ, and Toschi-Dias E contributed to acquisition and interpretation of the data, and technical procedures. Cunha PJ, Negrão CE, and Lorenzi-Filho G contributed to the scientific content of the manuscript. Ueno-Pardi LM was responsible for the design, data collection, and interpretation of intellectual and scientific content of the study, writing, review of the manuscript, and obtaining funding.

## Figures and Tables

**Figure 2A:**
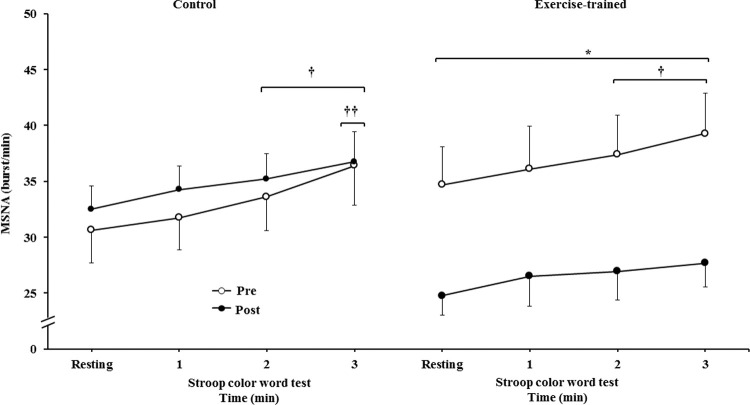
Muscle sympathetic nerve activity bursts frequency. * = Pre *versus* post at rest and during 3 min of SCWT in exercise-trained group, *p*<0.05; ^†^ = *versus* rest, *p*<0.05 (within group in pre and post intervention/control);^ ††^ = *versus* first and second min of the SCWT *p<*0.05 (within group in pre and post control). Values are mean ± SD. SCWT = Stroop color word test; MSNA = muscle sympathetic nerve activity.

**Figure 2B:**
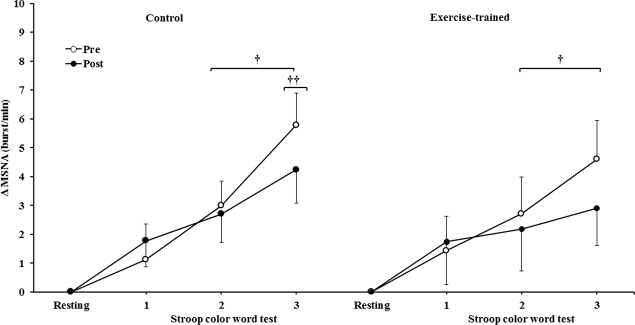
Muscle sympathetic nerve activity bursts frequency responses. ^†^ = *versus* rest, *p*<0.05 (within group in pre and post intervention/control);^ ††^ = *versus* first and second min of the SCWT *p<*0.05 (within group in pre and post control). Values are mean ± SD. SCWT = Stroop color word test; MSNA = muscle sympathetic nerve activity.

**Figure 5A:**
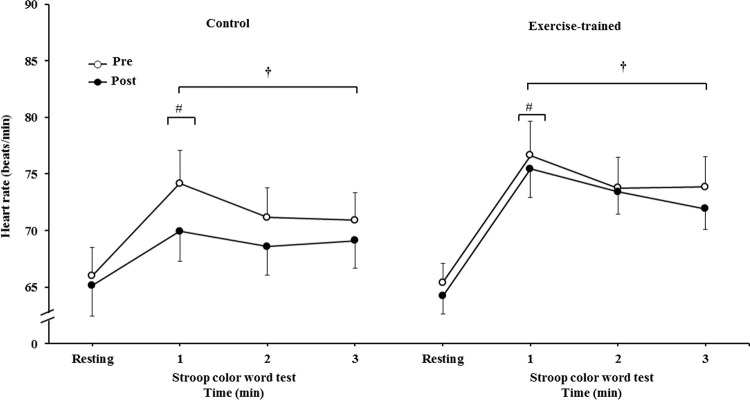
Heart rate at rest and during the SCWT. ^†^ = *versus* rest, *p*<0.05 (within group in pre and post intervention/control); ^#^ = *versus* second and third min of the SCWT *p<*0.05 (within group in within group in pre and post intervention/control). Values are mean ± SD. SCWT = Stroop Color Word Test

**Figure 5B:**
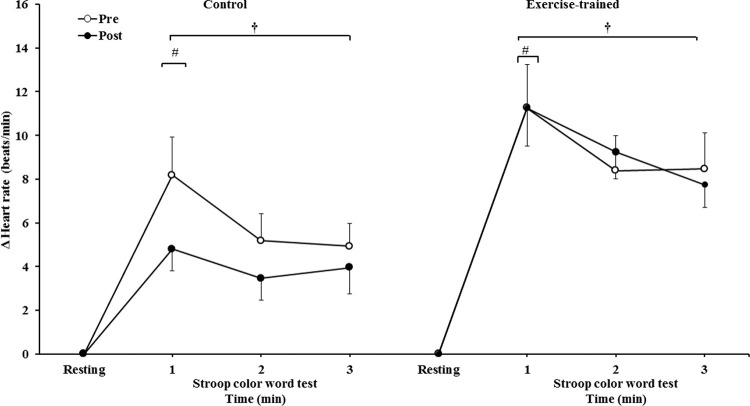
Heart rate responses during the SCWT. ^†^ = *versus* rest, *p*<0.05 (within group in pre and post intervention/control); ^#^ = *versus* second and third min of the SCWT *p<*0.05 (within group in within group in pre and post intervention/control). Values are mean ± SD. SCWT = Stroop Color Word Test.

**Figure 5C:**
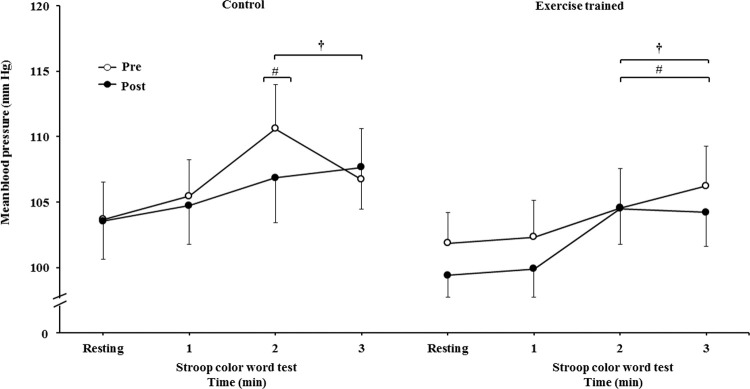
Ankle mean arterial BP at rest and during the SCWT.

**Figure 5D:**
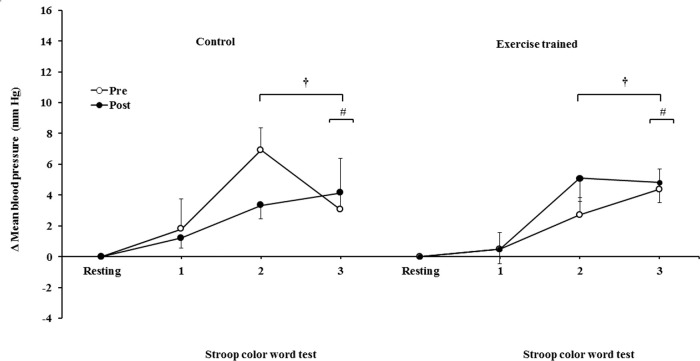
Ankle mean arterial BP responses during the SCWT. ^†^ = *versus* rest, *p*<0.05 (within group in pre and post intervention/control); ^#^ = *versus* first min of the SCWT *p<*0.05 (within group in pre and post intervention/control). Values are mean ± SD. BP = blood pressure; SCWT = Stroop Color Word Test.

**Table 1:** Baseline sociodemographic and clinical characteristics among the individuals with obstructive sleep apnea selected for control or exercise-trained groups.

	Control (n=15)	Exercise (n=18)	*p*
Physical characteristics			
Sex, female/male	5/10	9/9	0.46
Age, years	49.3±1.7	53.8±1.7	0.07
Body weight	83.20±3.6	81.51±3.8	0.75
BMI, kg/m^2^	29.7±0.9	29.3±0.9	0.77
ESS, score	12.7±1.4	11.3±1.4	0.50
Mental Status			
MEEM, score	27.7±0.5	27.1±0.6	0.44
Education, years	14.1±1.7	11.7±0.9	0.20
Estimated IQ, score	85.1±3.2	91.3±3.2	0.18
BAI, score	7.1±2.4	7.9±1.5	0.78
BDI, score	7.0±1.2	7.4±1.3	0.83
Metabolic parameters			
Fasting glucose, mg/Dl	98.9±2.9	103.2±1.6	0.18
Total Cholesterol, mg/dL	206.4±11.9	208.6±9.7	0.89
HDLc, mg/dL	44.1±3.2	48.6±2.2	0.24
LDLc, mg/Dl	130.5±12.1	136.1±8.2	0.70
Peak VO_2,_ mL/kg/min	24.2±1.3	24.6±1.3	0.83
Cardiovascular and neurovascular parameters			
Heart rate, beats/min	66.0±2.5	65.4±1.7	0.84
Systolic BP, mm Hg	122.9±3.1	121.3±2.4	0.69
Diastolic BP, mm Hg	79.9±2.3	78.6±1.5	0.62
MSNA, bursts/min	30.6±0.5	34.7±0.8	0.34
MSNA, bursts/100 heart beats	47.5±0.97	51.9±1	0.48
Sleep status			
Total sleep time, min	387.8±11.9	361.4±12.8	0.15
Sleep efficiency, %	84.7±1.7	81.2±2.4	0.25
AHI, events/h	39.8±5.5	45.8±7.8	0.55
Arousal index, events/h	28.3±2.3	33.9±4.2	0.27
O_2_ desaturation, events	29.1±5.3	41.0±7.7	0.23

Values are mean±SD. BMI = body mass index; ESS = Epworth sleepiness scale; MEEM = Mini-Mental state examination; IQ = intelligence quotient; BAI = Beck Anxiety Inventory; BDI = Beck Depression Inventory; HDLc = high-density lipoprotein cholesterol; LDLc = low-density lipoprotein cholesterol; Peak VO_2_ = peak oxygen consumption; BP = blood pressure; MSNA = muscle sympathetic nerve activity; AHI = apnea hypopnea index. There was no significant difference in sex, age, weight, BMI, body fat, Epworth sleepiness scale, Mini-Mental state examination, education, metabolic parameters, cardiovascular parameters, and sleep parameters among groups using non-paired *t* test. Sex was tested by using the χ^2^ test.

**Table 2:** Effects of exercise training on physical characteristics, physical capacity, and sleep parameters in individuals with obstructive sleep apnea.

	Pre	Post	Change
Physical characteristics			
Body weight, kg			
Control	83.2±3.6	84.3±3.6	1.1±0.4
Exercise	81.5±3.8	79.9±3.3*	-1.6±0.7^††^
BMI, kg/m^2^			
Control	29.7±0.9	29.9±0.9	0.2±0.3
Exercise	29.3±0.9	28.5±0.8*	-0.8±0.3^††^
Physical parameter			
Heart rate			
Control	66.0±2.5	65.0±2.7	-1.0±2.2
Exercise	65.4±1.7	64.2±1.6	-1.2±1.0
Systolic BP, mm Hg			
Control	122.9±3.1	122.2±3.9	-0.7±4.2
Exercise	121.3±2.4	114.1±2.9	-7.3±3.9
Diastolic BP, mm Hg			
Control	79.9±2.3	77.8±2.2	-2.1±3.0
Exercise	78.6±1.5	76.4±1.5	-2.1±2.3
VO_2_ peak_,_ mL.kg^-1^.min^-1^			
Control	24.2±1.3	23.0±1.5	-1.2±0.4
Exercise	24.6±1.3	29.3±1.5*^†^	4.7±0.8^††^
Sleep status			
Total sleep time, min			
Control	387.8±11.9	398.6±12.6	10.8±9.5
Exercise	361.4±12.8	370.2±12.7	8.8±12.3
Sleep efficiency, %			
Control	84.7±1.7	84.5±1.9	-0.2±2.0
Exercise	81.2±2.4	84.4±2.0	3.2±2.3
Arousal index, events/h			
Control	28.3±2.3	28.7±3.0	0.5±1.9
Exercise	33.9±4.2	26.3±3.3^†^	-7.6±3.0^††^
O_2_ desaturation, events			
Control	29.1±5.3	39.2±6.5	10.1±5.6
Exercise	41.0±7.7	34.1±6.1	-6.9±3.2^††^
AHI, events/h			
Control	39.8±5.5	45.3±6.9	5.5±3.3
Exercise	45.8±7.8	38.4±6.3	-7.4±3.1^††^

Values are means ± SD. BMI = body mass index; Peak VO_2_ = peak oxygen consumption; AHI = apnea-hypopnea index. * = Between group comparison, *p*<0.05; ^†^ = Within group comparisons post intervention, *p*<0.05 (2-way ANOVA); ^††^ = change between groups (Unpaired *t* test).
